# Seasonal variation in *Azumiobodo hoyamushi* infection among benthic organisms in the southern coast of Korea

**DOI:** 10.1186/s13071-015-1179-5

**Published:** 2015-11-04

**Authors:** Ki-Woong Nam, Yun-Kyung Shin, Kyung-Il Park

**Affiliations:** Department of Aquatic Life Medicine, College of Ocean Science and Technology, Kunsan National University, 558 Daehakno, Gunsan, 573-701 Republic of Korea; National Fisheries Research Institute, Busan, 619-705 Republic of Korea

**Keywords:** Benthic organisms, Soft tunic syndrome, Ascidiacea, *Halocynthia roretzi*, *Azumiobodo hoyamushi*

## Abstract

**Background:**

Recent studies have reported that soft tunic syndrome (STS) in the edible ascidian *Halocynthia roretzi* is caused by the kinetoplastid parasite *Azumiobodo hoyamushi*. In this study, we attempted to detect and quantify the pathogen in benthic animals.

**Methods:**

Four species of ascidians, three species of echinoderms, two species of bivalves, one species each of sponge and algae, as well as seawater, were collected in 2014 and 2015 from an ascidian farm on the southern coast of Korea by SCUBA diving. Samples were collected from ascidian hanging culture ropes or the sea bottom. Inhalent siphons were excised for the analysis of ascidians, and soft body tissues were excised from the other species. Membrane filters were used to filter collected seawater. Tissues and membrane filters were analysed using culture testing, PCR testing, and qPCR diagnoses.

**Results:**

Only organisms belonging to Ascidiacea are susceptible to *A. hoyamushi* infection*.* The infection rate (% infected of the total number collected) and infection intensity (number of cells infected/g tissue wet weight) varied depending on the seasonal variation in seawater temperatures. Most ascidians examined were infected with *A. hoyamushi* and showed higher infection intensity in cold water seasons (April 2014 and February 2015), followed by a dramatic drop during warm water seasons (August and November, 2014). In addition, infection intensity of *A. hoyamushi* during the warm water period was higher in ascidians from the sea bottom than those from the hanging culture rope.

**Conclusions:**

Among benthic organisms that inhabit the southern coast of Korea, most ascidians are susceptible to *A. hoyamushi* infection. Seasonal cycle of infection rates and intensities of the pathogen correspond well with the STS disappearance and onset cycle observed in ascidian farms. The high intensity of *A. hoyamushi* infection in the ascidians on the sea bottom of ascidian farms during summer suggest further studies on the role of the pathogen in resumption of STS occurrence in late fall or early winter in the southern coast of Korea.

## Background

*Halocynthia roretzi* represents an important species of Ascidiacea for the fishing industries of South Korea and Japan, and is produced by high-density culture [1, 2]. However, in the early 1990s, mass mortality of *H. roretzi* due to soft tunic syndrome (STS) occurred during winter and the following spring. Ascidian STS (AsSTS) has been occurring with comparable frequency in South Korea and Japan [3–6].

Kumagai et al. [7] isolated a flagellated parasite in STS-diseased *H. roretzi* cultured in Miyagi Prefecture in north eastern Japan and Hirose et al. [5] identified that flagellate as the causal agent of STS via challenge test, and named it as *Azumiobodo hoyamushi*. Meanwhile, Shin et al. [8] also reported flagellated parasites detected specifically in STS-diseased *H. roretzi* cultured in Tongyeong on the southern coast of South Korea. Kim et al. [9] identified these flagellated parasites as *A. hoyamushi* and indicated that AsSTS on the coasts of South Korea was caused by this pathogen, similar to Japan.

Kim et al. [9] reported that pure cultured *A. hoyamushi* thrived at a water temperature range of 10–15 °C, whereas temperatures outside this range caused reduced growth or death of *A. hoyamushi*. This indicates that water temperature is one of the factors that plays a major role in *A. hoyamushi* growth. Water temperatures along the southern coast of Korea vary greatly according to season, with the surface layer reaching more than 25 °C during summer (July to September) and falling below 10 °C during winter (January to February) [10]. Therefore, such water temperature differences are expected to induce seasonal variations in *A. hoyamushi* infection intensity in the tunics of *H. roretzi.*

In the present study, we collected a variety of benthic organisms that inhabit the waters around an ascidian farm during different seasons and quantitatively diagnosed *A. hoyamushi* in these organisms using molecular techniques. We examined the host range of *A. hoyamushi* and seasonal variations in the rate and intensity of infection by *A. hoyamushi*.

## Methods

### Sampling and analysis preparation

**Fig. 1:**
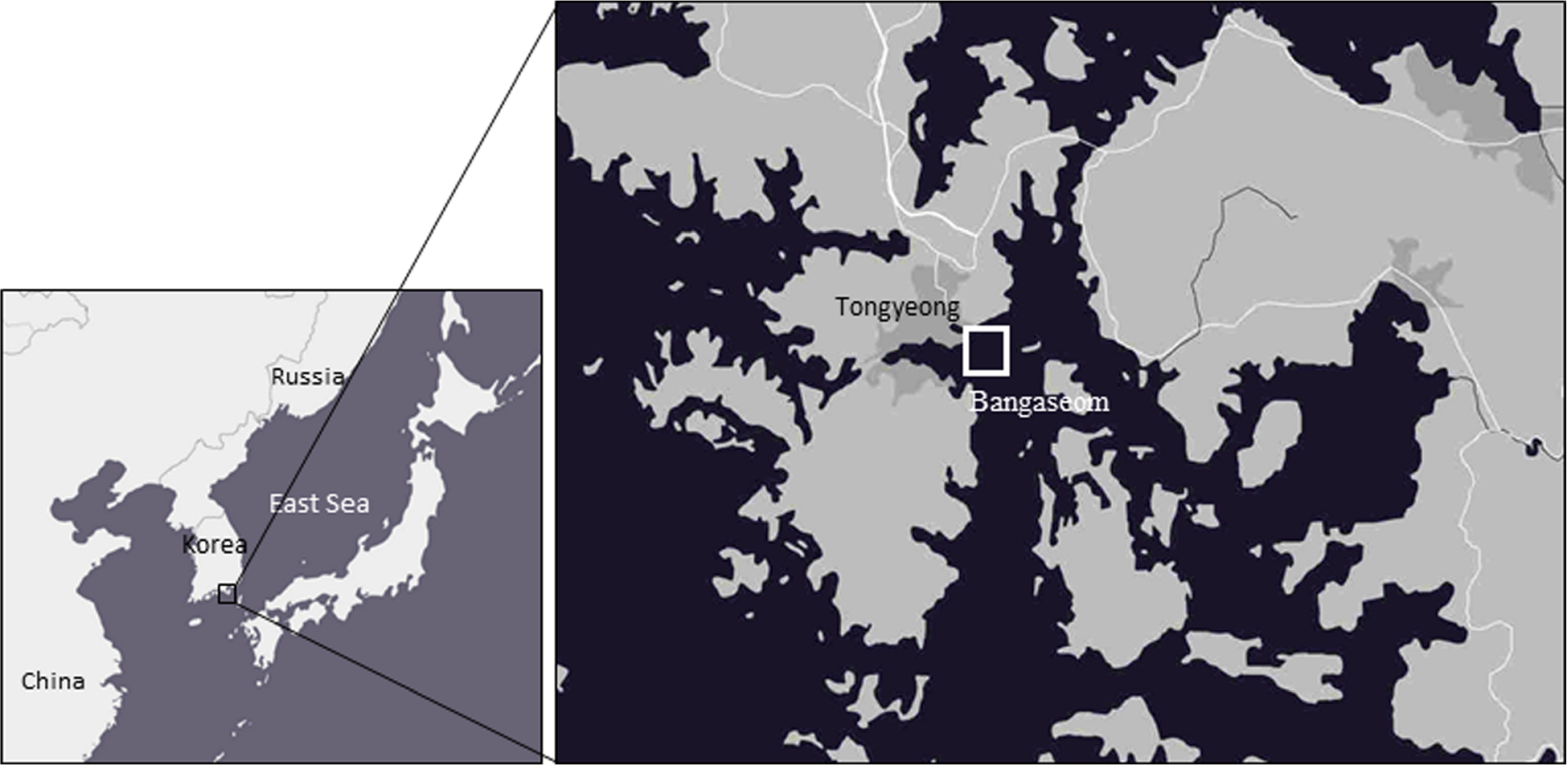
Sampling location on the southern coast of Korea

Organisms attached to ascidian hanging culture lines and benthic organisms on the bottom of the ascidian farm were collected by SCUBA diving during April, August, and November 2014 and February 2015 from Bangaseom ascidian farm located in Tongyeong (south Gyeonsang Province, South Korea) (Fig. 1). Seawater samples were also collected from the site at depths of 1 and 12 m during each collection period. Collected samples were transported to the laboratory for measurements of size and body weight. For ascidian organisms, incurrent siphons were excised for use in the analysis, while soft body tissues were excised from the other species. Membrane filters (Φ 0.2 μm, DISMIC-25AS, ADVANTEC, Japan) were used to filter 10 L of the collected seawater for 24 h. All tissues and membrane filters were divided into three equal parts by weight and each sample was used for culture testing, PCR testing, and qPCR diagnoses.

### Culture testing

Tissues excised from each sample were placed in minimum essential medium (MEM) and cultured for 5 days, followed by observation under a microscope to detect the presence of *A. hoyamushi*. Experimental procedures were performed in accordance with reported methodology [9].

### PCR test

DNA from the excised tissues was extracted using DNA blood and tissue kit (Qiagen, Korea). The primer used in the PCR diagnosis was F: 5′-GCC TCT GTG GTT TGC TCC TT-3′, R: 5′-TAC TGG GCG GCT TGG ATC TCG T-3′ [9], and the PCR cocktail was prepared by admixing 0.25 μL enzyme (5 units/μL, ExTaq™, TaKaRa, Japan), 5 μL buffer, 4 μL dNTP mixture, 100 ng DNA template, and 0.2 μM primer pair. Amplification of the *A. hoyamushi*-specific DNA sequence was performed using a PCR system (GeneAmp PCR system 9700, Applied Biosystems, USA). The PCR conditions consisted of pre-denaturation at 94 °C for 5 min, followed by 35 cycles of denaturation at 94 °C for 1 min, annealing at 64 °C for 30 s, and extension at 72 °C for 1 min. For the PCR results, amplified products of 642 bp were confirmed by electrophoresis on a 1 % agarose gel.

### qPCR

After pulverization of 150 mg of each sample, DNA was extracted using DNA blood kit (Qiagen) and *A. hoyamushi* was quantified using the Taq-Man probe technique [11]. The primer used in the qPCR was F: 5′-GCC TCT GTG GTT TGC TCC TT-3′, R: 5′-TAC TGG GCG GCT TGG ATC TCG T-3′ and the probe was 5′ FAM-CCG CTC AAA GAC GAA CTA CAG CGA -BHQ1 3′. Preparation of the qPCR cocktail consisted of adding template DNA, primer pair, probe, and distilled water to the premix (Bioneer, Korea) to bring it to a final volume of 20 μL, followed by amplification of the *A. hoyamushi*-specific DNA sequence using Exicycler™96 (Bioneer). The qPCR conditions were as follows: pre-denaturation at 94 °C for 5 min, followed by 45 cycles of denaturation at 94 °C for 1 min, annealing at 58 °C for 30 s, and extension at 72 °C for 1 min, after which the reaction results were observed. Infection intensity was tested according to reported methodology [11]. Briefly, in vitro cultured *A. hoyamushi* was diluted to 1, 10, 100, 1000, and 100,000 cells and the Ct value for each parasite concentration was obtained using real-time PCR. After deriving the correlation equation for the parasite concentration and Ct value, the Ct value confirmed from real-time PCR for the sample in question was substituted into this equation to quantify the results, which were expressed as the number of cells/g tissue wet weight.

## Results

### Samples

Samples included four species of ascidians, three species of echinoderms, two species of bivalves, one species of sponge, and one species of algae (Table 1). This distribution of species was similar for each sampling period. Ascidians attached to the ascidian hanging culture rope were identified as *H. roretzi*, *Styela clava* and *Pyura vittata.* Ascidians collected from the bottom of the ascidian farm were *H. roretzi*, *S. clava*, *S. plicata* and *P. vittata.* In addition to these, echinoderms *Asterias amurensis, A. pectinifera* and *Luidia quinaria* and bivalves *Mytilus galloprovincialis* and *Atrina pectinata* were collected from the bottom of the ascidian farm. One species each of sponge and algae were also collected. Water temperatures at 1 m depth in 2014 were 15, 25 and 19 °C in April, August, and November, respectively, and dropped to 9 °C in February 2015. Water temperatures at 12 m depth in 2014 were 15, 21 and 19 °C in April, August, and November, respectively, and dropped to 9 °C in February 2015. These water temperatures were almost within the range of variation in water temperature for each month recorded by KOOFS during the same period as our study [12].

**Table 1:** Infection rates of *Azumiobodo hoyamushi* among benthic organisms on the southern coast of Korea, measured by cultivation of tissues in minimal essential media (MEM)

Specimens		Sampling period			
Tunicates	Collection source	Apr. 2014	Aug.	Nov.	Feb. 2015
*Halocynthia roretzi*	Hanging rope	30 %(3/10)	20 %(2/10)	20 %(2/10)	50 %(5/10)
*Styela clava*	Hanging rope	20 %(1/5)	–	40 %(4/10)	60 %(3/5)
*Pyura vittata*	Hanging rope	–	25 %(1/4)	100 %(2/2)	50 %(1/3)
*Halocynthia roretzi*	Bottom	–	50 %(5/10)	20 %(2/10)	20 %(2/10)
*Styela clava*	Bottom	0 %(0/5)	30 %(3/10)	0 %(0/5)	60 %(3/5)
*Styela plicata*	Bottom	–	25 %(1/4)	0 %(0/5)	60 %(3/5)
*Pyura vittata*	Bottom	–	100 %(2/2)	–	50 %(1/2)
Echinoderms					
*Asterias amurensis*	Bottom	0 %(0/5)	0 %(0/5)	0 %(0/5)	0 %(0/5)
*Asterina pectinifera*	Bottom	0 %(0/5)	0 %(0/1)	0 %(0/5)	0 %(0/5)
*Luidia quinaria*	Bottom	–	0 %(0/5)	–	0 %(0/1)
Bivalves					
*Mytilus galloprovincialis*	Bottom	0 %(0/5)	0 %(0/5)	0 %(0/5)	0 %(0/2)
*Atrina pectinata*	Bottom	–	0 %(0/3)	–	0 %(0/1)
Sponge					
Unknown	Bottom	–	–	0 %(0/5)	0 %(0/5)
Algae					
*Undaria pinnatifida*	Bottom	0 %(0/5)	0 %(0/1)	-	0 %(0/1)
Seawater					
Seawater	Surface	100 %(1/1)	0 %(0/1)	0 %(0/1)	0 %(0/1)
	Bottom	100 %(1/1)	0 %(0/1)	0 %(0/1)	0 %(0/1)

### Culture test

*Azumiobodo hoyamushi* infection, confirmed via a culture test, was found only in ascidians and was not detected in the echinoderms, bivalves, sponge, or algae (Table 1). Confirmed *A. hoyamushi* infection in *H. roretzi* on the ascidian hanging culture rope was detected throughout the investigation period, with infection rates decreasing from 30 % in April to 20 % in August and November, then rising to 50 % the following February. In August, 50 % of *H. roretzi* collected from the sea bottom were infected, decreasing to 20 % in November and the following February. Among other ascidians collected from the bottom of the ascidian farm, 0–20 % were infected during April and November 2014, increasing to 20–60 % the following February. In seawater samples, *A. hoyamushi* was detected only in April.

### PCR testing

The PCR test also revealed *A. hoyamushi* infection only in ascidians, similar to the culture test. Seasonal variation in infection rates showed an overall pattern of abundance in April, decreasing in August, increasing again in November, and maintaining the rate until the following February (Table 2). In April, 90 % of *H. roretzi* from hanging culture ropes were infected. In August, 20 % were infected, followed by an increase to 80 % in November, which was then maintained at about 60 % until the following February. *Halocynthia roretzi* was not collected from the sea bottom in April; however, about 50 % of those collected in August were infected, whereas in November and the following February, *A. hoyamushi* infection was found in all individuals. In samples of *S. clava* attached to the hanging culture rope, 40–100 % were infected. In *S. clava* samples collected from the bottom of the ascidian farm, no individual was infected in April, 30 % were infected in August, and 60 % were infected in November as well as in the following February. Most of the *P. vittata* individuals attached to the hanging culture ropes and those inhabiting the sea bottom were also infected.

**Table 2:** Infection rates of *Azumiobodo hoyamushi* among benthic organisms on the southern coast of Korea, measured by PCR

Specimens		Sampling period			
	Collection source	Apr. 2014	Aug.	Nov.	Feb. 2015
Tunicates					
*Halocynthia roretzi*	Hanging rope	90 %(9/10)	20 %(2/10)	80 %(8/10)	60 %(6/10)
*Styela clava*	Hanging rope	100 %(5/5)	–	40 %(4/10)	60 %(3/5)
*Pyura vittata*	Hanging rope	–	25 %(1/4)	100 %(2/2)	100 %(3/3)
*Halocynthia roretzi*	Bottom	–	50 %(5/10)	60 %(6/10)	60 %(6/10)
*Styela clava*	Bottom	0 %(0/5)	30 %(3/10)	60 %(3/5)	60 %(3/5)
*Styela plicata*	Bottom	–	50 %(2/4)	60 %(3/5)	80 %(4/5)
*Pyura vittata*	Bottom	–	100 %(2/2)	–	50 % (1/2)
Echinoderms					
*Asterias amurensis*	Bottom	0 %(0/5)	0 %(0/5)	0 %(0/5)	0 %(0/5)
*Asterina pectinifera*	Bottom	0 %(0/5)	0 %(0/1)	0 %(0/5)	0 %(0/5)
*Luidia quinaria*	Bottom	–	0 %(0/5)	–	0 %(0/1)
Bivalves					
*Mytilus galloprovincialis*	Bottom	0 %(0/5)	0 %(0/5)	0 %(0/5)	0 %(0/2)
*Atrina pectinata*	Bottom	–	0 %(0/3)	–	0 %(0/1)
Sponge					
Unknown	Bottom	–	–	0 %(0/5)	0 %(0/5)
Algae					
*Undaria pinnatifida*	Bottom	0 %(0/5)	0 %(0/1)	-	0 %(0/1)
Seawate**r**					
Seawater	Surface	100 %(1/1)	0 %(0/1)	0 %(0/1)	0 %(0/1)
	Bottom	100 %(1/1)	0 %(0/1)	0 %(0/1)	0 %(0/1)

### qPCR

**Fig. 2:**
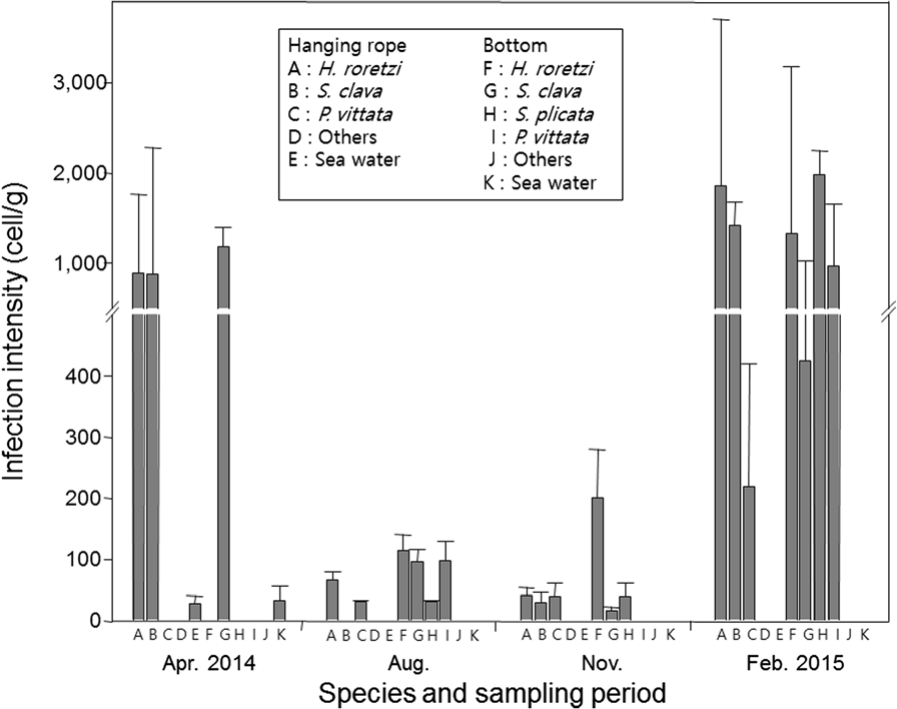
Seasonal variation in infection intensity of *Azumiobodo hoyamushi* among benthic organisms on the southern coast of Korea, measured by qPCR

Quantification of *A. hoyamushi* using real-time PCR showed that infection intensity in *H. roretzi* from the rope culture averaged to 889.13 cells/g in April. The infection intensity decreased rapidly to 66.25 cells/g by August, continued to decrease to 41.70 cells/g in November, then drastically increased to 1858.07 cells/g by the following February (Fig. 2). Infection intensity of *A. hoyamushi* in *H. roretzi* found on the bottom of the ascidian farm was 115.12 cells/g in August and 201.10 cells/g in November, increasing rapidly to 1323.55 cells/g by the following February. The changes in infection intensity in *S. clava* and *P. vittata* were similar to those in *H. roretzi*. Samples of *S. plicata* were collected only from the bottom of the ascidian farm and *A. hoyamushi* infection intensities in August and November were similar to those of other ascidians. However, in February 2015, 1984.38 cells/g were recorded. In seawater, infection intensities of *A. hoyamushi* were 28.37 cells/g at 1 m depth and 32.72cells/g at 12 m depth.

## Discussion

In the present study, we investigated the prevalence and infection intensity of *A. hoyamushi* using tissue culture method and molecular techniques. In particular, the inhalent tissue of ascidians was removed and used for quantification of the pathogen. Shin et al. [11] examined the infection intensity of *A. hoyamushi* in various parts of the tunic, including inhalent siphon, exhalent siphon, and three different parts of the tunic; they found that the inhalent siphon was the most sensitive organ for the diagnosis of the pathogen. They also reported that infection intensity of *A. hoyamushi* increased as STS developed and the pathogens spread to other parts of the tunic from the inhalent siphon. Hirose et al. [12] also reported that the inhalent siphon of *H. roretzi* is the point of entry of *A. hoyamushi*. Therefore, the inhalent siphon tissue is regarded as the best tissues for the quantitative measurement of *A. hoyamushi* in ascidians.

Approximately 20 species of ascidians have been reported to inhabit the waters of South Korea, of which five species are edible [2, 13]. As observed in the present study, *A. hoyamushi* infections were confirmed in *H. roretzi*, *S. clava, S. plicata*, and *P. vittata*. With the exception of *P. vittata*, these species are produced commercially on the southern coast of Korea by high-density culture. In most of these species, *A. hoyamushi* infection rates exceeded 50 % in the PCR test results and infection intensities were confirmed to reach tens to thousands of individuals per gram of host tissue. With such broad occurrence in ascidians, the high infection rates and intensities discovered in this study are proposed to enhance opportunities for interspecific *A. hoyamushi* infection within ascidians and are believed to be the cause of the continued occurrence of AsSTS in ascidian farms. Kumagai et al. [14] also reported that *S. clava* is a potential carrier of *A. hoyamushi* in ascidians. According to a recent report [15], mass death of *S. clava* has been seen during summer seasons in Tongyeong, with the primary symptom being softening of the body, a symptom that is similar to AsSTS. Generally, AsSTS is not known to occur frequently during summer seasons [16]. However, given that symptoms seen during *S. clava* mass death were similar to those of STS, and that *A. hoyamushi* infections occur during summer, as confirmed in the present study, investigations of the influence of *A. hoyamushi* on *S. clava* mass death should be undertaken.

Seasonal variation in *A. hoyamushi* infection rates showed a pattern of being low during summer and increasing during winter or spring. A more prominent seasonal variability was seen in infection intensity, reaching 1000 cells/g or more in *H. roretzi*, *S. clava*, and *P. vittata* in April 2014 and decreasing to less than 100 cells/g in August. Such patterns continued until November; however, by the following February, infection intensity increased to the level of the previous April. Seasonal changes in *A. hoyamushi* infection intensity in *H. roretzi* appear to be based on seasonal variations in water temperature. According to Kim et al. [9], *A. hoyamushi* showed the highest growth rate at 15 °C, and conversely, at temperatures below 10 °C or above 20 °C, growth reduced or led to death. The relationship between water temperature and *A. hoyamushi* infection intensity was similar to that between water temperature and *A. hoyamushi* growth reported previously [9]. Therefore, the present study strongly suggests that the year-round variation in *A. hoyamushi* infection intensity in *H. roretzi* from ascidian farms is determined by water temperature. In ascidian farms, AsSTS disappears during summer seasons and reoccurs during early winter seasons [2, 17]. Thus, it appears that *A. hoyamushi* infection intensity decreases as water temperature rises in summer, whereas infection intensity increases as water temperature drops.

*Azumiobodo hoyamushi* infection rate and intensity also varied according to water depth. Infection rate and intensity were drastically higher in wild ascidians inhabiting the seabed of the ascidian farm (water depth of 12 m) during summer (August), when STS decreased, than in ascidians attached to hanging culture rope (water depth of 1 m). This is likely because most of the parasites on the surface layer had died, given that the water temperature on the surface layer had reached 25 °C at the time of collection of samples. The temperature at the bottom layer of the ascidian farm reached only 21 °C, a lower water temperature is associated with a lower *A. hoyamushi* mortality rate, leading to the survival of more number of parasites than that in the upper layer. Thus, conditions in the bottom layer enabled *A. hoyamushi* to survive inside ascidian organisms inhabiting the bottom, although STS disappeared from ascidians in the hanging culture ropes due to the high water temperature. It is suggested that surviving parasites in ascidians on the sea bottom were then able to infect new hosts attached to ascidian hanging culture ropes when the water temperature dropped in winter. Meanwhile, Nawata et al. [18] suggested the role of cysts of the pathogen. According to the study, cyst-like cells of *A. hoyamushi* can survive on the sea bottom at high temperatures, followed by excystment in the host at low water temperatures.

## Conclusions

Among benthic organisms that inhabit the southern coast of Korea, *A. hoyamushi* was detected only in ascidians, and most of the ascidians sampled were infected by *A. hoyamushi*. Infection rates and intensities showed a seasonal cycle of reduction during summer, when the water temperature is high, and abundance during seasons when the water temperature is low. Infection rates and intensities also varied according to water depth. It is believed that warmer surface temperatures led to death of the parasites, whereas deeper, cooler water allowed survival of a number of parasites in the ascidians at the bottom of the sea. High occurrence of *A. hoyamushi* on the sea bottom suggest additional studies on the role of the pathogens for resumption of STS occurrence in late fall or early winter on the southern coast of Korea.
